# Differential Regenerative Capacity of the Optic Tectum of Adult Medaka and Zebrafish

**DOI:** 10.3389/fcell.2021.686755

**Published:** 2021-06-29

**Authors:** Yuki Shimizu, Takashi Kawasaki

**Affiliations:** ^1^Functional Biomolecular Research Group, Biomedical Research Institute, National Institute of Advanced Industrial Science and Technology, Osaka, Japan; ^2^DBT-AIST International Laboratory for Advanced Biomedicine, National Institute of Advanced Industrial Science and Technology, Osaka, Japan

**Keywords:** radial glia, stab wound injury, optic tectum, neuronal differentiation, reactive gliosis, zebrafish, medaka

## Abstract

Zebrafish have superior regenerative capacity in the central nervous system (CNS) compared to mammals. In contrast, medaka were shown to have low regenerative capacity in the adult heart and larval retina, despite the well-documented high tissue regenerative ability of teleosts. Nevertheless, medaka and zebrafish share similar brain structures and biological features to those of mammals. Hence, this study aimed to compare the neural stem cell (NSC) responses and regenerative capacity in the optic tectum of adult medaka and zebrafish after stab wound injury. Limited neuronal differentiation was observed in the injured medaka, though the proliferation of radial glia (RG) was induced in response to tectum injury. Moreover, the expression of the pro-regenerative transcriptional factors ascl1a and oct4 was not enhanced in the injured medaka, unlike in zebrafish, whereas expression of sox2 and stat3 was upregulated in both fish models. Of note, glial scar-like structures composed of GFAP^+^ radial fibers were observed in the injured area of medaka at 14 days post injury (dpi). Altogether, these findings suggest that the adult medaka brain has low regenerative capacity with limited neuronal generation and scar formation. Hence, medaka represent an attractive model for investigating and evaluating critical factors for brain regeneration.

## Introduction

Zebrafish have a superior ability to regenerate various tissues, including the central nervous system (CNS) and heart, compared with mammals (Becker et al., 1997; Poss et al., 2002; Raymond et al., 2006; März et al., 2011). Recently, to better understand the molecular mechanisms underlying the high regenerative capacity of zebrafish, comparative analyses of tissue regeneration in the retina and heart between zebrafish and mice have been performed, given their similarities in cell type and tissue structure (Kang et al., 2016; Hoang et al., 2020; Simões et al., 2020). Comparative studies using next-generation sequencing technology have revealed differences in the immune response or expression of transcriptional factors associated with tissue regeneration (Hoang et al., 2020; Simões et al., 2020). In contrast, the brain structure and cell types between zebrafish and mice are quite different (Kizil et al., 2012; Alunni and Bally-Cuif, 2016; Diotel et al., 2020; Labusch et al., 2020). Despite the efforts made to explore and compare the brain regeneration mechanisms in zebrafish and mice, comparative studies with omics approaches have not been well examined (Llorens-Bobadilla et al., 2015; Zhong et al., 2016; Arneson et al., 2018; Yu and He, 2019; Demirci et al., 2020). To investigate the mechanisms that contribute to the high regenerative capacity of the zebrafish brain, non-regenerative animal models with similar brain structures and biological features are warranted.

Medaka (*Oryzias latipes*) is a popular experimental model among freshwater teleosts that has been extensively used for tissue regeneration analysis. Despite its high regenerative capacity in the fin and pancreas (Akimenko et al., 1995; Katogi et al., 2004; Moss et al., 2009; Otsuka and Takeda, 2017), similar to zebrafish, medaka have a low capacity for heart and retina regeneration (Ito et al., 2014; Lai et al., 2017; Lust and Wittbrodt, 2018). Comparative analysis of heart regeneration between adult medaka and zebrafish, cardiac cryoinjury results in less cardiomyocyte proliferation and scar formation in medaka (Ito et al., 2014; Lai et al., 2017), whereas zebrafish show induced cardiomyocyte proliferation and injured tissues are filled with newborn cardiomyocytes, with little or no scar tissue formation (Poss et al., 2002; Kikuchi and Poss, 2012). Regenerative capacity in the retina has also been compared between larval medaka and zebrafish, indicating that retinal injury induces Müller glia proliferation in both models; however, Müller glia in medaka have less multipotency, with photoreceptors being generated, but not retinal ganglion cells (Lust and Wittbrodt, 2018). Moreover, overexpression of sox2 in Müller glia was found to promote the regenerative potential of these cells in the medaka retina. However, the CNS regenerative capacity in the adult medaka remains unclear.

Medaka and zebrafish have similar brain structures and niches of adult neural stem cells (NSCs) (Adolf et al., 2006; Grandel et al., 2006; Alunni et al., 2010; Kuroyanagi et al., 2010). Stab wound injury models affecting various regions of the adult zebrafish brain, including the optic tectum, have been developed to investigate brain regeneration (Kroehne et al., 2011; März et al., 2011; Kishimoto et al., 2012; Kaslin et al., 2017; Shimizu et al., 2018; Lindsey et al., 2019; Yu and He, 2019). The optic tectum of both zebrafish and medaka harbors two types of NSCs—neuroepithelial-like stem (NE) and radial glia (RG) cells—that express stem cell markers, such as *sox2* and *msi1*. NE cells are proliferative cells that produce neurons, RG, and oligodendrocytes, whereas most of RG are quiescent (Alunni et al., 2010; Ito et al., 2010; Takeuchi and Okubo, 2013; Galant et al., 2016; Dambroise et al., 2017). Previous studies showed that RG proliferation and differentiation into newborn neurons are induced in response to injury in young adult zebrafish (2–4 months old) (Shimizu et al., 2018; Ueda et al., 2018; Yu and He, 2019; Kiyooka et al., 2020). In contrast, the regenerative responses in the medaka tectum remain to be elucidated.

Herein, the proliferation and differentiation of RG and NE in injured medaka and zebrafish were examined to evaluate the regenerative capacity of the medaka brain. The present study highlights the potential of medaka as a useful experimental non-regenerative model to investigate and identify pro-regenerative factors that mediate CNS regeneration.

## Materials and Methods

### Animals

Medaka (*O. latipes*) and zebrafish (*Danio rerio*), specifically the Kyoto-Cab and RIKEN Wako wild-type strains, respectively, were maintained at 27.0 ± 1°C under a 14/10 h light/dark cycle. All experimental protocols were approved by the Institutional Animal Care and Use Committee of the National Institute of Advanced Industrial Science and Technology (2021-0276). Animals with 3–7 months old were used for all experiments, except for the analysis of newborn neurons after tectum injury, which 3–5-months-old medaka and zebrafish were used.

### Stab Wound Injury Protocol

To induce a stab wound injury in the adult optic tectum, medaka and zebrafish were anesthetized with 0.02% tricaine (pH 7.0; Nacalai Tesque, Kyoto, Japan) and a 30 G needle was vertically inserted into the medial region of the right hemisphere, as previously described (Shimizu et al., 2018). The contralateral uninjured hemisphere was used as internal control for each animal. For quantitative real-time polymerase chain reaction (PCR) analysis, both hemispheres were injured.

### 5-Bromo-2-Deoxyuridine (BrdU) Administration

To label proliferating cells, injured medaka and zebrafish were kept in 5 mM BrdU (Wako, Osaka, Japan). Injured medaka and zebrafish were treated with BrdU for 48 h, from 1 to 3 days post injury (dpi).

### Histological and Immunohistochemical Analysis

Medaka and zebrafish were anesthetized using 0.02% tricaine and intracardially perfused with phosphate-buffered saline. Brains were dissected and stored in 4% paraformaldehyde (Wako) solution overnight at 4°C. The fixed brains were stored in 30% sucrose solution overnight at 4°C, and whole brains were then embedded in a 2:1 mixture of 30% sucrose and Tissue-Tek O.C.T. compound (Sakura Finetek Japan, Tokyo, Japan). For fluorescence immunohistochemistry, 14 μm cryosections were prepared using a Leica CM1960 cryostat (Leica Biosystems, Wetzlar, Germany). Fluorescence immunohistochemistry was performed as described previously, using the following primary antibodies: mouse anti-HuC (1:100 dilution, A21271; Invitrogen, Waltham, MA, United States) as a pan-neuronal marker, mouse anti-proliferating cell nuclear antigen (PCNA) (1:200, sc-56; Santa Cruz Biotechnology, Dallas, TX, United States) as a proliferating cell marker, mouse anti-glial fibrillary acid protein (GFAP) (1:500, G3893; Sigma-Aldrich, St. Louis, MO, United States), and rabbit anti-brain lipid binding protein (BLBP) (1:500, ABN14; Millipore, Burlington, MA, United States) as RG cell markers, and sheep anti-BrdU (1:500, ab1893; Abcam, Cambridge, United Kingdom). Alexa Fluor 488- and 546-conjugated subclass-specific antibodies (1:500, Invitrogen) were used as secondary antibodies. For PCNA antigen retrieval, sections were incubated with 10 mM sodium citrate for 30 min at 85°C prior to primary antibody incubation. For BrdU antigen retrieval, sections were incubated with 2N HCl (Wako) for 30 min at 37°C. For nuclear staining, the sections were incubated with Hoechst 33258 (1:500; Dojindo, Kumamoto, Japan) for 30 min following immunohistochemistry.

### Quantitative Real-Time PCR (qRT-PCR)

For qRT-PCR, both hemispheres of the optic tectum were injured. After anesthesia with 0.02% tricaine, both hemispheres of the optic tectum were dissected from one fish and homogenized in TRIzol reagent (Invitrogen). Total RNA was purified using the Directozol RNA Miniprep (Zymo Research, Irvine, CA, United States), and cDNA was synthesized using RevaTra Ace (Toyobo, Osaka, Japan). The gene-specific primers used for *ascl1a, oct4, sox2, stat3*, and *tbp* are listed in Supplementary Table 1. The expression of tbp was used as endogenous control.

### Cell Quantification

To quantify proliferating RG after the stab injury, the number of BLBP^+^PCNA^+^ cells was counted in 5–10 sections, including the center of the injury. To quantify NE proliferation, the number of PCNA^+^ cells located in the tectal marginal zone was counted in 5–10 sections, including the center of the injury. To quantify the number of newborn neurons after the stab injury, the number of BrdU^+^HuC^+^ cells in five sections, including the center of the injury, was counted. The number of BrdU^+^HuC^+^ cells in the tectal marginal zone was also counted in five sections after the tectum injury. The corresponding contralateral regions were examined as internal controls.

### Statistical Analysis

All data are expressed as the mean ± standard error of the mean (SEM), and sample numbers are indicated in each figure legend. Statistical analysis in two experimental groups was performed using paired and unpaired Student’s *t*-tests. In three or more groups, one-way analysis of variance was performed, followed by Tukey’s *post hoc* test. *P*-values were calculated using Prism software (GraphPad Software, San Diego, CA, United States) and statistical significance was defined as ^∗∗∗^ and ††† if *P* < 0.001; ^∗∗^ and †† if *P* < 0.01; ^∗^ and † if *P* < 0.05.

## Results

### Increase in the Proliferation of Radial Glia in Response to Stab Injury

**FIGURE 1:**
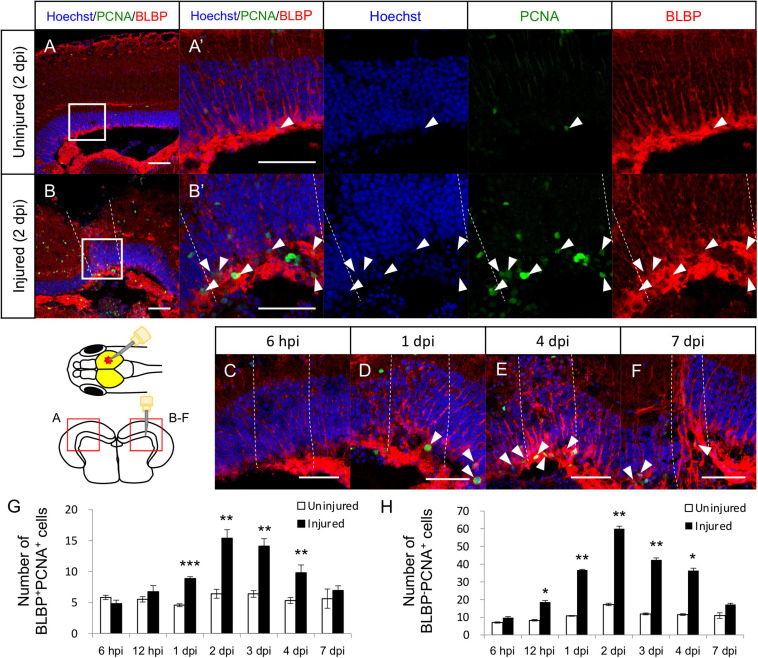
Proliferation of radial glia (RG) is increased in response to stab wound injury. Representative images of proliferative RG (BLBP^+^PCNA^+^ cells) in the uninjured **(A)** and injured **(B)** hemispheres at 2 days post injury (dpi). **(A’,B’)** Magnified images of the boxed area in **(A,B)**. **(C–F)** Representative images of proliferative RG in the injured hemisphere at 6 h post injury (hpi) and at 1, 4, and 7 dpi. White arrowheads indicate BLBP^+^PCNA^+^ cells, and dashed lines indicate injured areas. Scale bar: 50 μm in **(A–F)** and (**A’**,**B’**). Schematic drawing of the stab injury in the right hemisphere of the optic tectum and cross-section. **(G)** Quantification of proliferative RG in both uninjured and injured hemispheres at 6 (*n* = 5) and 12 (*n* = 3) hpi, and 1 (*n* = 5), 2 (*n* = 5), 3 (*n* = 4), 4 (*n* = 5), and 7 (*n* = 4) dpi. **(H)** Quantification of proliferative cells (BLBP^–^PCNA^+^ cells) except NE in both uninjured and injured hemispheres at 6 (*n* = 5) and 12 (*n* = 3) hpi, and 1 (*n* = 5), 2 (*n* = 5), 3 (*n* = 4), 4 (*n* = 5), and 7 (*n* = 4) dpi. Statistical analyses between uninjured and injured hemispheres at each time point were evaluated using paired Student’s *t*-tests. Statistical significance was defined as *P < 0.05; **P < 0.01; ***P < 0.001.

In the adult zebrafish optic tectum, most RG are quiescent under physiological conditions, but stab wound injury can induce their proliferation (Shimizu et al., 2018; Lindsey et al., 2019; Yu and He, 2019). To examine that this regenerative mechanism was also present in medaka, stab wound injury was induced in the right hemisphere of the optic tectum of medaka and RG proliferation was quantified by counting BLBP (RG marker), and PCNA (proliferating cell marker) double-positive cells. At 2 dpi, the number of proliferative RG cells (BLBP^+^PCNA^+^ cells) was significantly increased in the injured hemisphere than in the contralateral (internal control) uninjured side (Figures 1A,B). Additional analysis between 6 h post injury (hpi) to 7 dpi (Figures 1C–F) further revealed that the number of proliferative RG significantly increased from 1 dpi and peaking at around 2 dpi, with no significant difference being observed at 7 dpi (Figure 1G), which follows the same response trend observed in the injured zebrafish (Shimizu et al., 2018; Yu and He, 2019). Moreover, we quantified BLBP^–^PCNA^+^ cells except for proliferative NE known as PCNA^+^ cells located in the tectal marginal zone to analyze the cell proliferation of another type of cell. These BLBP^–^PCNA^+^ cells which may include oligodendrocytes, microglia, neutrophils, and endothelial cells also significantly increased in response to the injury (Figure 1H). Although the contribution of NE to tectum regeneration is controversial (Shimizu et al., 2018; Lindsey et al., 2019), NE proliferation after stab wound injury was also evaluated by counting the PCNA^+^ cells in the tectal marginal zone (Supplementary Figures 1A–L). This analysis confirmed that the stab wound injury had no significant effect on the proliferation of NE (Supplementary Figure 1M), which is consistent with previous injured zebrafish (Shimizu et al., 2018). Taken together, these results suggest that RG in the medaka and zebrafish tectum have similar proliferative potential after injury.

### Limited Generation of Newborn Neurons After Stab Injury of Optic Tectum

**FIGURE 2:**
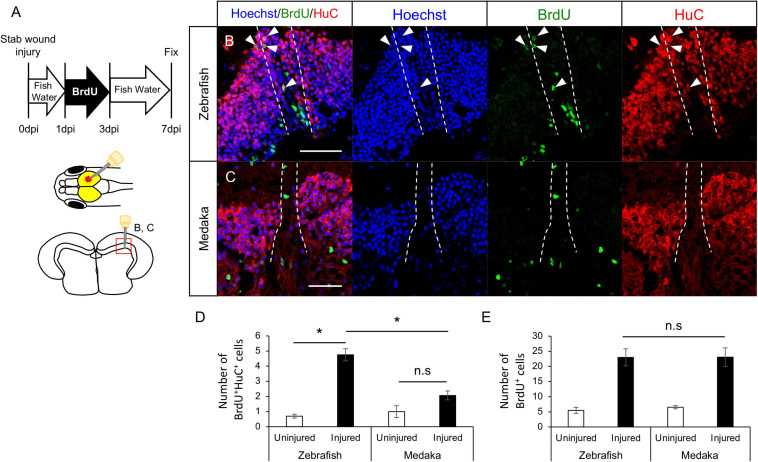
Generation of newborn neurons in the injured medaka is limited compared with zebrafish. **(A)** Schematic drawing of bromodeoxyuridine (BrdU)-labeling and stab injury in the right hemisphere of the optic tectum. Representative images of newborn neurons (BrdU^+^HuC^+^ cells) in both injured zebrafish **(B)** and medaka **(C)**. White arrowheads indicate BrdU^+^HuC^+^ cells, and dashed lines indicate the injured areas. Scale bar: 50 μm. **(D)** Quantification of BrdU^+^HuC^+^ cells in the uninjured and injured hemispheres in zebrafish (*n* = 4) and medaka (*n* = 4). **(E)** Quantification of total BrdU^+^ cells in both uninjured and injured zebrafish (*n* = 4) and medaka (*n* = 4). Statistical analyses were evaluated using one-way analysis of variance followed by Tukey’s *post hoc* test. Statistical significance was defined as *P < 0.05.

Previous studies showed that newborn neurons around the injured site after the tectum injury in young adult zebrafish are mainly derived from RG (Shimizu et al., 2018; Yu and He, 2019). To analyze whether newborn neurons were similarly generated in tectum injured medaka, BrdU-labeled proliferative cells (including RG and NE) in the injured zebrafish and medaka were evaluated at 7 dpi (Figure 2A). We confirmed that RG incorporated BrdU at 3 dpi (Supplementary Figures 2A–E). Then, the number of newborn neurons (BrdU^+^HuC^+^ cells) at 7 dpi was quantified (Figures 2B,C), revealing that were not significantly increased in the injured hemisphere in the medaka unlike in the zebrafish (Figure 2D). BrdU^+^ cells around the injured periventricular gray zone (PGZ) in the medaka optic tectum are BLBP^+^ (Supplementary Figures 2F–I). Moreover, the number of BrdU^+^ cells observed in PGZ was not significantly different in both fish models (Figure 2E). These results suggest that post-proliferating RG in injured medaka have limited capacity for neuronal differentiation. As NE can also generate neuronal cells in the optic tectum, the differentiation potential of BrdU^+^ cells in the tectal marginal zone after tectum injury was also evaluated (Supplementary Figure 3A). However, no significant differences were observed in the BrdU^+^HuC^+^ cells around the tectal marginal zone between injured and uninjured hemispheres in both medaka and zebrafish (Supplementary Figures 3B–G). Overall, these results suggest that post-proliferating RG in the injured medaka tectum have limited neuronal differentiation, whereas stab wound injury in the optic tectum does not affect NE differentiation into neurons.

### Differential Expression of Transcriptional Factors Between Medaka and Zebrafish After Tectum Injury

**FIGURE 3:**
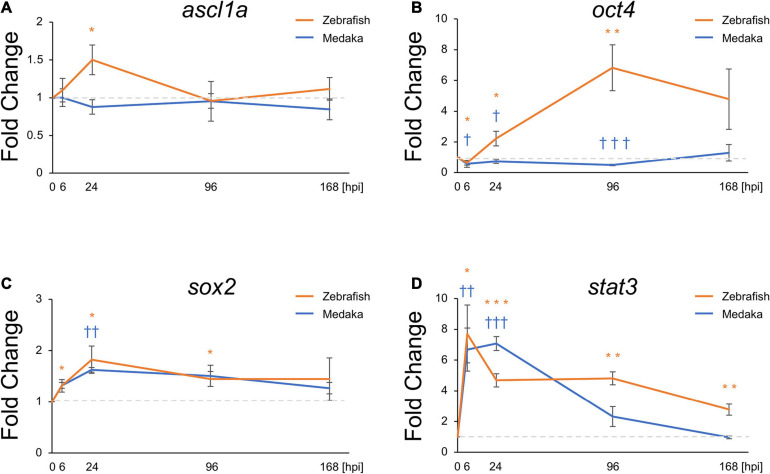
Pro-regenerative transcriptional factors are differentially expressed between the injured medaka and zebrafish. Quantitative polymerase chain reaction analysis of the pro-regenerative transcriptional factors ascl1a **(A)**, oct4 **(B)**, sox2 **(C)**, and stat3 **(D)**. Graphs indicate the relative gene expression in the injured tectum from 6 to 168 h post injury (hpi) compared to the uninjured tectum (*n* = 4). Statistical analyses between the uninjured and injured hemispheres at each time point were evaluated by unpaired Student’s *t*-tests. We used * for zebrafish and ^†^ for medaka to indicate significant difference. Statistical significance was defined as * and ^†^*P* < 0.05; ** and ^††^*P* < 0.01; *** and ^†††^ *P* < 0.001.

Molecular mechanisms related to *ascl1a* during zebrafish retina regeneration have been well studied (Fausett et al., 2008; Ramachandran et al., 2010). In particular, the expression of this pro-regenerative transcriptional factor was shown to be induced the optic tectum of zebrafish. Moreover, induction of *sox2, stat3*, and *oct4* expression was also shown to be required for NSC proliferation and differentiation into neurons (Fausett et al., 2008; Ramachandran et al., 2010; Nelson et al., 2012; Zhao et al., 2014; Gorsuch et al., 2017; Sharma et al., 2019). Herein, the expression of these transcriptional factors was also evaluated to assess potential changes induced in response to the tectum injury. Thus, *ascl1a, oct4* (*pou5f1* in medaka and *pou5f3* in zebrafish), *sox2*, and *stat3* were evaluated at 6, 24, 96, and 168 hpi. Expression changes of *sox2* and *stat3* showed similar patterns, significantly increasing from 6 hpi (Figures 3A,B). At 168 hpi, *stat3* expression in zebrafish remained significantly elevated though *stat3* expression in medaka returned to baseline. Interestingly, upregulation of *ascl1a* and *oct4* was observed in the injured zebrafish (Figures 3C,D), whereas it was not induced in the injured medaka. Expression of *oct4* was decreased at 6 hpi in both injured medaka and zebrafish, subsequently increasing in the injured zebrafish at 24 and 96 hpi, but not in the injured medaka (Figure 3D). These results suggest that differential expression of pro-regenerative factors may contribute for the limited neuronal differentiation potential of RG in medaka during tectum regeneration.

### Glial Scar-Like Structures Persist in the Injured Medaka Tectum

**FIGURE 4:**
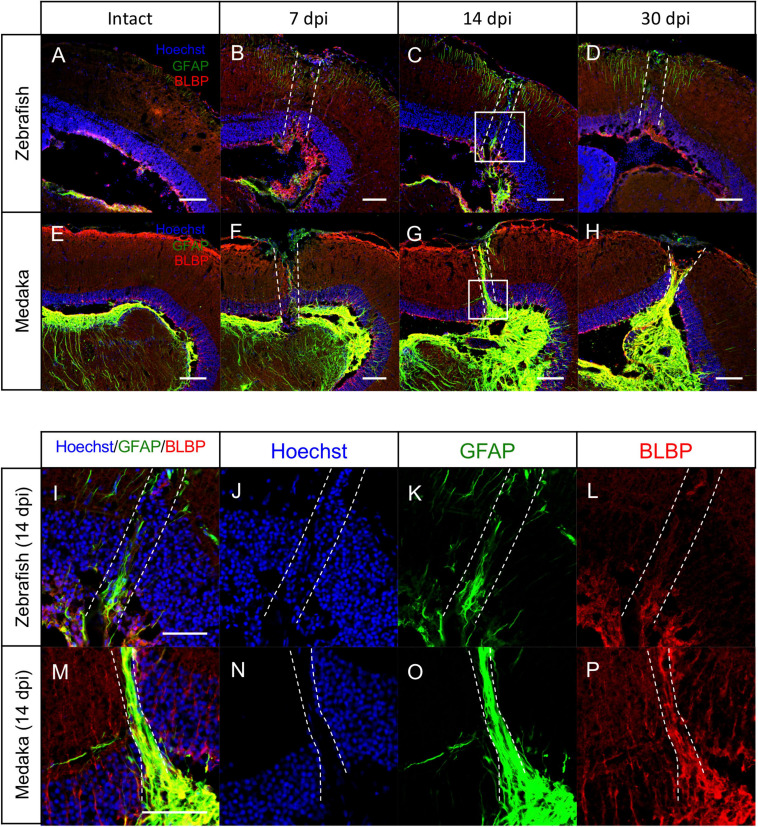
Persistent glial scar-like structure is observed in the injured medaka tectum. **(A–H)** Representative images of immunostaining with anti-GFAP and anti-BLBP antibodies on the uninjured (**A:** zebrafish and **B:** medaka) and injured hemisphere at 7, 14, and 30 days post injury (dpi) [**(B–D)**: zebrafish and **(E–H)**: medaka]. **(I–P)** Magnified images of the boxed area at 14 dpi [**(I–L)**: zebrafish and **(M–P)**: medaka]. The dashed lines indicate the injured areas. Scale bar: 100 **(A–H)** and 50 **(I,M)** μm.

In the adult mammalian brain, stab wound injury increases GFAP immunoreactivity in astrocytes, called reactive gliosis, and these reactive astrocytes are shown to contribute to the GFAP^+^ scar formation, called glial scar (Feeney et al., 1981; Hozumi et al., 1990; Smith et al., 1995; Xiong et al., 2013; Burda et al., 2016). Although stab wound injury in the zebrafish telencephalon also increases GFAP immunoreactivity in the injured hemisphere, scar formation has not been observed (Kroehne et al., 2011; März et al., 2011; Baumgart et al., 2012; Kishimoto et al., 2012). Hence, the reactive gliosis after the tectum injury was herein assessed by comparing GFAP immunoreactivity in injured medaka and zebrafish at 7, 14, and 30 dpi (Figures 4A–H). At 7 dpi, GFAP expression increased in both injured fishes (Figures 4B,F). In particular, the GFAP immunoreactivity remained activated in the injured zebrafish at 14 dpi (Figures 4I–P), compared with the uninjured tectum (Figure 4C); however, its levels were relatively weak and no obvious scar-like structure was observed at 30 dpi (Figure 4D). Surprisingly, GFAP^+^ scar-like structures were formed in the injured medaka at 14 dpi (Figure 4G), which persisted at 30 dpi (Figure 4H). Moreover, at 14 dpi (Figures 4I–P), the injured medaka lacked cell layer in the injured PGZ indicated by dashed lines (Figure 4N), and GFAP^+^ fibers covered the area of this missing cell layer (Figure 4O). This GFAP^+^ scar-like structure elongated from the basal layer of the PGZ to the apical side (Figure 4O) and this injury-induced GFAP^+^ structures were co-expressed with BLBP (Figures 4O,P), suggesting that RG in the medaka optic tectum could form this scar-like structure in response to injury. Of note, in the injured zebrafish optic tectum, a disturbed cell layer due to the injury was also observed (Figure 4J), but no obvious lack of layer and no accumulation of GFAP^+^ or BLBP^+^ radial fibers around the injured area were noted (Figures 4J–L). These results suggest that RG with GFAP^+^ scar-like structures in the injured medaka tectum have reactive astrocytic characteristics.

## Discussion

Zebrafish have higher CNS regeneration capacity, including of the brain, retina, and spinal cord, compared with mammals (Kizil et al., 2012; Alunni and Bally-Cuif, 2016). Medaka and zebrafish share similar biological features, such as brain structure, body size, and lifespan; nevertheless, medaka have different regenerative capacities in heart and retina (Ito et al., 2014; Lai et al., 2017; Lust and Wittbrodt, 2018). The present study showed that stab wound injury could induce the proliferation of RG in the medaka, but with limited generation of newborn neurons in the injured site compared with the response observed in similarly injured zebrafish. Therefore, this is the first report indicating the limited capacity of neuronal regeneration in the teleost young adult brain. We also confirmed that there was no induction of transcriptional factors, *ascl1a* and *oct4* in the injured medaka. Moreover, we observed injury-induced GFAP^+^ radial fibers from RG at 14 dpi and found that this glial scar-like structure covered the injured area with lack of cell layer in medaka. Taken together, our findings suggest that medaka have low regenerative ability in the tectum compared to zebrafish because RG in the injured medaka tectum may have reactive astrocytic characteristics rather than neurogenic NSCs.

In the adult zebrafish CNS, the optic tectum and retina share similar features regarding NSCs. For example, RG in the optic tectum and Müller glia in the retina are quiescent under physiological conditions, whereas proliferation and differentiation of these NSCs are activated upon injury (Raymond et al., 2006; Ito et al., 2010; Shimizu et al., 2018). The optic tectum and retina also have NE cells that continuously proliferate and generate newborn neurons throughout life (Raymond et al., 2006; Ito et al., 2010). Comparative analyses of retinal regeneration showed that Müller glia in the larval medaka have limited neuronal differentiation compared with larval zebrafish despite the proliferative response induced by retinal injury (Lust and Wittbrodt, 2018), which is consistent with here observed limited capacity of RG in the medaka tectum. Furthermore, although Müller glia in the medaka only contribute for the generation of photoreceptors, induction of *sox2* expression in Müller glia after retinal injury can restore their multi-potency. Although *sox2* and *stat3* expression increased in both medaka and zebrafish after the tectum injury, that of *ascl1a* and *oct4* did not increase in the injured medaka. In the zebrafish, the transcriptional factors ascl1a (also known as Ascl1/Mash1 in mammals) and *oct4* are known to play important roles in retinal regeneration (Fausett et al., 2008; Ramachandran et al., 2010; Sharma et al., 2019). Furthermore, during zebrafish retinal regeneration from light damage, *stat3* expression may precede *ascl1a* expression (Nelson et al., 2012; Goldman, 2014), whereas N-methyl-*d*-asparate-injured mouse retina showed the lack of *Ascl1* expression (Karl et al., 2008) despite the upregulation of phosphorylated Stat3 (Jorstad et al., 2020). These findings suggest that upregulation of STAT3-mediated signaling is a shared feature in both injured medaka and zebrafish, but that lack of *ascl1a* expression in the injured medaka may result in low neurogenic capacity of RG in the medaka tectum.

In addition to limited neuronal generation after medaka tectum injury, persistent GFAP^+^BLBP^+^ scar-like structures were clearly observed from 14 to 30 dpi. In contrast, in the zebrafish adult brain, stab wound injury in the telencephalon induced reactive gliosis with upregulation of GFAP immunoreactivity, but no scar formation was observed (März et al., 2011; Baumgart et al., 2012; Kishimoto et al., 2012). In the injured zebrafish optic tectum, although upregulation of GFAP immunoreactivity was also observed, obvious scar formation like medaka has not been observed. These findings suggest that scar-like structures with radial fibers in the injured medaka tectum are similar to glial scar formed by reactive astrocytes in the damaged mammalian CNS (Burda et al., 2016). Glial scar in the injured rodent CNS includes GFAP and other extracellular matrices, such as chondroitin sulfate proteoglycan and collagen IV (McKeon et al., 1991). The role of glial scar in the tissue regeneration is well investigated, but the findings remain inconclusive (Anderson et al., 2016; Adams and Gallo, 2018; Yang et al., 2020). Glial scar is shown to prevent acute inflammation spreading; however, large scar is an obstacle for neuronal and axonal regeneration. Whether glial scar-like structure in the injured medaka shares these features remain to be explored. Furthermore, Stat3 activation in astrocytes is involved in glial scar formation after spinal cord injury in mice (Herrmann et al., 2008; Anderson et al., 2016), suggesting that the activated stat3 signaling in the medaka RG may contribute to scar formation rather than neuronal generation unlike zebrafish.

Teleost species are shown to have a high regenerative capacity of various tissues, including the CNS. In addition to zebrafish, goldfish (*Carassius auratus*) and brown ghost knifefish (*Apteronotus leptorhynchus*) are known to have high CNS regeneration potential (Bernstein, 1964; Stevenson and Yoon, 1978; Zupanc, 1999; Sîrbulescu et al., 2009). In addition to these teleosts, recently, various other species including salmonoids (masu and chum salmon) (Pushchina et al., 2017, 2020) and killifish (mummichog, *Aphaniops hormuzensis*, and *Nothobranchius furzeri*) (Bisese et al., 2019; Soltani et al., 2020; Van houcke et al., 2021) have been explored as models to assess the mechanisms regulating the CNS regenerative potential. Previous studies showed that only medaka have low CNS regenerative potential, regardless of age and health condition (Lust and Wittbrodt, 2018). For comparative analyses of tissue regeneration, compatible injury models and similar biological properties, except regenerative capacity, are important. Hence, medaka represent an attractive non-regenerative model to investigate and identify pro-regenerative factors that mediate CNS regeneration.

## Data Availability Statement

The original contributions presented in the study are included in the article/Supplementary Material. Further inquiries can be directed to the corresponding author/s.

## Ethics Statement

The animal study was reviewed and approved by the Institutional Animal Care and Use Committee at the National Institute of Advanced Industrial Science and Technology.

## Author Contributions

YS and TK designed the experiments and wrote and revised the manuscript. YS performed histological and molecular experiments. Both authors approved the submitted version of the manuscript.

## Conflict of Interest

The authors declare that the research was conducted in the absence of any commercial or financial relationships that could be construed as a potential conflict of interest.

## References

[B1] Adams K. L. Gallo V. (2018). The diversity and disparity of the glial scar. *Nat. Neurosci.* 21 9–15. 10.1038/s41593-017-0033-9 29269757 PMC5937232

[B2] Adolf B. Chapouton P. Lam C. S. Topp S. Tannhauser B. Strahle U. (2006). Conserved and acquired features of adult neurogenesis in the zebrafish telencephalon. *Dev. Biol.* 295 278–293. 10.1016/j.ydbio.2006.03.023 16828638

[B3] Akimenko M. A. Johnson S. L. Westerfield M. Ekker M. (1995). Differential induction of four msx homeobox genes during fin development and regeneration in zebrafish. *Development* 121 347–357. 7768177 10.1242/dev.121.2.347

[B4] Alunni A. Bally-Cuif L. (2016). A comparative view of regenerative neurogenesis in vertebrates. *Development* 143 741–753. 10.1242/dev.122796 26932669 PMC4813331

[B5] Alunni A. Hermel J. M. Heuzé A. Bourrat F. Jamen F. Joly J. S. (2010). Evidence for neural stem cells in the medaka optic tectum proliferation zones. *Dev. Neurobiol.* 70 693–713. 10.1002/dneu.20799 20506557

[B6] Anderson M. A. Burda J. E. Ren Y. Ao Y. O’Shea T. M. Kawaguchi R. (2016). Astrocyte scar formation aids central nervous system axon regeneration. *Nature* 532 195–200. 10.1038/nature17623 27027288 PMC5243141

[B7] Arneson D. Zhang G. Ying Z. Zhuang Y. Byun H. R. Ahn I. S. (2018). Single cell molecular alterations reveal target cells and pathways of concussive brain injury. *Nat. Commun.* 9:3894. 10.1038/s41467-018-06222-0 30254269 PMC6156584

[B8] Baumgart E. V. Barbosa J. S. Bally-Cuif L. Götz M. Ninkovic J. (2012). Stab wound injury of the zebrafish telencephalon: a model for comparative analysis of reactive gliosis. *Glia* 60 343–357. 10.1002/glia.22269 22105794

[B9] Becker T. Wullimann M. F. Becker C. G. Bernhardt R. R. Schachner M. (1997). Axonal regrowth after spinal cord transection in adult zebrafish. *J. Comp. Neurol.* 377 577–595. 9007194 10.1002/(sici)1096-9861(19970127)377:4<577::aid-cne8>3.0.co;2-#

[B10] Bernstein J. J. (1964). Relation of spinal cord regeneration to age in adult goldfish. *Exp. Neurol.* 1964 161–174. 10.1016/0014-4886(64)90014-7 14126124

[B11] Bisese E. C. Ciuba C. M. Davidson A. L. Kaushik A. Mullen S. M. Barth J. L. (2019). The acute transcriptome response of the midbrain/diencephalon to injury in the adult mummichog (*Fundulus heteroclitus*). *Mol. Brain* 12:119. 10.1186/s13041-019-0542-4 31888716 PMC6937918

[B12] Burda J. E. Bernstein A. M. Sofroniew M. V. (2016). Astrocyte roles in traumatic brain injury. *Exp. Neurol.* 275 305–315. 10.1016/j.expneurol.2015.03.020 25828533 PMC4586307

[B13] Dambroise E. Simion M. Bourquard T. Bouffard S. Rizzi B. Jaszczyszyn Y. (2017). Postembryonic fish brain proliferation zones exhibit neuroepithelial-type gene expression profile. *Stem Cells* 35 1505–1518. 10.1002/stem.2588 28181357

[B14] Demirci Y. Cucun G. Poyraz Y. K. Mohammed S. Heger G. Papatheodorou I. (2020). Comparative transcriptome analysis of the regenerating zebrafish telencephalon unravels a resource with key pathways during two early stages and activation of wnt/β-catenin signaling at the early wound healing stage. *Front. Cell Dev. Biol.* 8:584604. 10.3389/fcell.2020.584604 33163496 PMC7581945

[B15] Diotel N. Lübke L. Strähle U. Rastegar S. (2020). Common and distinct features of adult neurogenesis and regeneration in the telencephalon of zebrafish and mammals. *Front. Neurosci.* 14:568930. 10.3389/fnins.2020.568930 33071740 PMC7538694

[B16] Fausett B. V. Gumerson J. D. Goldman D. (2008). The proneural basic helix-loop-helix gene ascl1a is required for retina regeneration. *J. Neurosci.* 28 1109–1117. 10.1523/JNEUROSCI.4853-07.2008 18234889 PMC2800945

[B17] Feeney D. M. Boyeson M. G. Linn R. T. Murray H. M. Dail W. G. (1981). Responses to cortical injury: I. Methodology and local effects of contusions in the rat. *Brain Res.* 211 67–77. 10.1016/0006-8993(81)90067-6 7225844

[B18] Galant S. Furlan G. Coolen M. Dirian L. Foucher I. Bally-Cuif L. (2016). Embryonic origin and lineage hierarchies of the neural progenitor subtypes building the zebrafish adult midbrain. *Dev. Biol.* 420 120–135. 10.1016/j.ydbio.2016.09.022 27693369 PMC5156517

[B19] Goldman D. (2014). Müller glial cell reprogramming and retina regeneration. *Nat. Rev. Neurosci.* 15 431–442. 10.1038/nrn3723 24894585 PMC4249724

[B20] Gorsuch R. A. Lahne M. Yarka C. E. Petravick M. E. Li J. Hyde D. R. (2017). Sox2 regulates Muller glia reprogramming and proliferation in the regenerating zebrafish retina via Lin28 and Ascl1a. *Exp. Eye Res.* 161 174–192. 10.1016/j.exer.2017.05.012 28577895 PMC5554723

[B21] Grandel H. Kaslin J. Ganz J. Wenzel I. Brand M. (2006). Neural stem cells and neurogenesis in the adult zebrafish brain: origin, proliferation dynamics, migration and cell fate. *Dev. Biol.* 295 263–277. 10.1016/j.ydbio.2006.03.040 16682018

[B22] Herrmann J. E. Imura T. Song B. Qi J. Ao Y. Nguyen T. K. (2008). STAT3 is a critical regulator of astrogliosis and scar formation after spinal cord injury. *J. Neurosci.* 28 7231–7243. 10.1523/JNEUROSCI.1709-08.2008 18614693 PMC2583788

[B23] Hoang T. Wang J. Boyd P. Wang F. Santiago C. Jiang L. (2020). Gene regulatory networks controlling vertebrate retinal regeneration. *Science* 370:eabb8598. 10.1126/science.abb8598 33004674 PMC7899183

[B24] Hozumi I. Chiu F. C. Norton W. T. (1990). Biochemical and immunocytochemical changes in glial fibrillary acidic protein after stab wounds. *Brain Res.* 524 64–71. 10.1016/0006-8993(90)90492-t 2400932

[B25] Ito K. Morioka M. Kimura S. Tasaki M. Inohaya K. Kudo A. (2014). Differential reparative phenotypes between zebrafish and medaka after cardiac injury. *Dev. Dyn.* 243 1106–1115. 10.1002/dvdy.24154 24947076

[B26] Ito Y. Tanaka H. Okamoto H. Ohshima T. (2010). Characterization of neural stem cells and their progeny in the adult zebrafish optic tectum. *Dev. Biol.* 342 26–38. 10.1016/j.ydbio.2010.03.008 20346355

[B27] Jorstad N. L. Wilken M. S. Todd L. Finkbeiner C. Nakamura P. Radulovich N. (2020). STAT signaling modifies Ascl1 chromatin binding and limits neural regeneration from muller glia in adult mouse retina. *Cell Rep.* 30 2195–2208. 10.1016/j.celrep.2020.01.075 32075759 PMC7148114

[B28] Kang J. Hu J. Karra R. Dickson A. L. Tornini V. A. Nachtrab G. (2016). Modulation of tissue repair by regeneration enhancer elements. *Nature* 532 201–206. 10.1038/nature17644 27049946 PMC4844022

[B29] Karl M. O. Hayes S. Nelson B. R. Tan K. Buckingham B. Reh T. A. (2008). Stimulation of neural regeneration in the mouse retina. *Proc. Natl. Acad. Sci. U.S.A.* 105 19508–19513. 10.1073/pnas.0807453105 19033471 PMC2614791

[B30] Kaslin J. Kroehne V. Ganz J. Hans S. Brand M. (2017). Distinct roles of neuroepithelial-like and radial glia-like progenitor cells in cerebellar regeneration. *Development* 144 1462–1471. 10.1242/dev.144907 28289134

[B31] Katogi R. Nakatani Y. Shin-i T. Kohara Y. Inohaya K. Kudo A. (2004). Large-scale analysis of the genes involved in fin regeneration and blastema formation in the medaka *Oryzias latipes*. *Mech. Dev.* 121 861–872. 10.1016/j.mod.2004.03.015 15210191

[B32] Kikuchi K. Poss K. D. (2012). Cardiac regenerative capacity and mechanisms. *Annu. Rev. Cell Dev. Biol.* 28 719–741. 10.1146/annurev-cellbio-101011-155739 23057748 PMC3586268

[B33] Kishimoto N. Shimizu K. Sawamoto K. (2012). Neuronal regeneration in a zebrafish model of adult brain injury. *Dis. Model Mech.* 5 200–209. 10.1242/dmm.007336 22028327 PMC3291641

[B34] Kiyooka M. Shimizu Y. Ohshima T. (2020). Histone deacetylase inhibition promotes regenerative neurogenesis after stab wound injury in the adult zebrafish optic tectum. *Biochem. Biophys. Res. Commun.* 529 366–371. 10.1016/j.bbrc.2020.06.025 32703437

[B35] Kizil C. Kaslin J. Kroehne V. Brand M. (2012). Adult neurogenesis and brain regeneration in zebrafish. *Dev. Neurobiol.* 72 429–461. 10.1002/dneu.20918 21595047

[B36] Kroehne V. Freudenreich D. Hans S. Kaslin J. Brand M. (2011). Regeneration of the adult zebrafish brain from neurogenic radial glia-type progenitors. *Development* 138 4831–4841. 10.1242/dev.072587 22007133

[B37] Kuroyanagi Y. Okuyama T. Suehiro Y. Imada H. Shimada A. Naruse K. (2010). Proliferation zones in adult medaka (*Oryzias latipes*) brain. *Brain Res.* 1323 33–40. 10.1016/j.brainres.2010.01.045 20114034

[B38] Labusch M. Mancini L. Morizet D. Bally-Cuif L. (2020). Conserved and divergent features of adult neurogenesis in zebrafish. *Front. Cell Dev. Biol.* 8:525. 10.3389/fcell.2020.00525 32695781 PMC7338623

[B39] Lai S. L. Marín-Juez R. Moura P. L. Kuenne C. Lai J. K. H. Tsedeke A. T. (2017). Reciprocal analyses in zebrafish and medaka reveal that harnessing the immune response promotes cardiac regeneration. *eLife* 6:e25605. 10.7554/eLife.25605 28632131 PMC5498136

[B40] Lindsey B. W. Aitken G. E. Tang J. K. Khabooshan M. Douek A. M. Vandestadt C. (2019). Midbrain tectal stem cells display diverse regenerative capacities in zebrafish. *Sci. Rep.* 9:4420. 10.1038/s41598-019-40734-z 30872640 PMC6418144

[B41] Llorens-Bobadilla E. Zhao S. Baser A. Saiz-Castro G. Zwadlo K. Martin-Villalba A. (2015). Single-cell transcriptomics reveals a population of dormant neural stem cells that become activated upon brain injury. *Cell Stem Cell* 17 329–340. 10.1016/j.stem.2015.07.002 26235341

[B42] Lust K. Wittbrodt J. (2018). Activating the regenerative potential of Müller glia cells in a regeneration-deficient retina. *eLife* 7:e32319. 10.7554/eLife.32319 29376827 PMC5815849

[B43] März M. Schmidt R. Rastegar S. Strähle U. (2011). Regenerative response following stab injury in the adult zebrafish telencephalon. *Dev. Dyn.* 240 2221–2231. 10.1002/dvdy.22710 22016188

[B44] McKeon R. J. Schreiber R. C. Rudge J. S. Silver J. (1991). Reduction of neurite outgrowth in a model of glial scarring following CNS injury is correlated with the expression of inhibitory molecules on reactive astrocytes. *J. Neurosci.* 11 3398–3411. 10.1523/JNEUROSCI.11-11-03398.1991 1719160 PMC6575543

[B45] Moss J. B. Koustubhan P. Greenman M. Parsons M. J. Walter I. Moss L. G. (2009). Regeneration of the pancreas in adult zebrafish. *Diabetes* 58 1844–1851. 10.2337/db08-0628 19491207 PMC2712797

[B46] Nelson C. M. Gorsuch R. A. Bailey T. J. Ackerman K. M. Kassen S. C. Hyde D. R. (2012). Stat3 defines three populations of Muller glia and is required for initiating maximal muller glia proliferation in the regenerating zebrafish retina. *J. Comp. Neurol.* 520 4294–4311. 10.1002/cne.23213 22886421 PMC3478445

[B47] Otsuka T. Takeda H. (2017). Targeted ablation of pancreatic beta cells in medaka. *Zoolog. Sci.* 34 179–184. 10.2108/zs170004 28589841

[B48] Poss K. D. Wilson L. G. Keating M. T. (2002). Heart regeneration in zebrafish. *Science* 298 2188–2190. 10.1126/science.1077857 12481136

[B49] Pushchina E. V. Kapustyanov I. A. Varaksin A. A. (2020). Neural Stem cells/neuronal precursor cells and postmitotic neuroblasts in constitutive neurogenesis and after, traumatic injury to the mesencephalic tegmentum of juvenile chum salmon, oncorhynchus keta. *Brain Sci.* 10:65. 10.3390/brainsci10020065 31991815 PMC7071460

[B50] Pushchina E. V. Zharikova E. I. Varaksin A. A. (2017). Persistent and reparative neurogenesis in the juvenile masu salmon *Oncorhynchus masou* telencephalon after mechanical injury. *Russ. J. Dev. Biol.* 48 307–320.

[B51] Ramachandran R. Fausett B. V. Goldman D. (2010). Ascl1a regulates Müller glia dedifferentiation and retinal regeneration through a Lin-28-dependent, let-7 microRNA signalling pathway. *Nat. Cell Biol.* 2010 1101–1107. 10.1038/ncb2115 20935637 PMC2972404

[B52] Raymond P. A. Barthel L. K. Bernardos R. L. Perkowski J. J. (2006). Molecular characterization of retinal stem cells and their niches in adult zebrafish. *BMC Dev. Biol.* 6:36. 10.1186/1471-213X-6-36 16872490 PMC1564002

[B53] Sharma P. Gupta S. Chaudhary M. Mitra S. Chawla B. Khursheed M. A. (2019). Oct4 mediates Muller glia reprogramming and cell cycle exit during retina regeneration in zebrafish. *Life Sci. Alliance* 2:e201900548. 10.26508/lsa.201900548 31594822 PMC6784428

[B54] Shimizu Y. Ueda Y. Ohshima T. (2018). Wnt signaling regulates proliferation and differentiation of radial glia in regenerative processes after stab injury in the optic tectum of adult zebrafish. *Glia* 66 1382–1394. 10.1002/glia.23311 29411422

[B55] Simões F. C. Cahill T. J. Kenyon A. Gavriouchkina D. Vieira J. M. Sun X. (2020). Macrophages directly contribute collagen to scar formation during zebrafish heart regeneration and mouse heart repair. *Nat. Commun.* 11:600. 10.1038/s41467-019-14263-2 32001677 PMC6992796

[B56] Sîrbulescu R. F. Iliȩ I. Zupanc G. K. (2009). Structural and functional regeneration after spinal cord injury in the weakly electric teleost fish, *Apteronotus leptorhynchus*. *J. Comp. Physiol. Neuroethol. Sens. Neural Behav. Physiol.* 195 699–714. 10.1007/s00359-009-0445-4 19430939

[B57] Smith D. H. Soares H. D. Pierce J. S. Perlman K. G. Saatman K. E. Meaney D. F. (1995). A model of parasagittal controlled cortical impact in the mouse: cognitive and histopathologic effects. *J. Neurotrauma* 12 169–178. 10.1089/neu.1995.12.169 7629863

[B58] Soltani A. R. Motamedi M. Teimori A. (2020). Adult neuronal regeneration in the telencephalon of the killifish *Aphaniops hormuzensis*. *J. Exp. Zool. B Mol. Dev. Evol.* 334 350–361. 10.1002/jez.b.23008 33107185

[B59] Stevenson J. A. Yoon M. G. (1978). Regeneration of optic nerve fibers enhances cell proliferation in the goldfish optic tectum. *Brain Res.* 153 345–351. 10.1016/0006-8993(78)90413-4 687985

[B60] Takeuchi A. Okubo K. (2013). Post-proliferative immature radial glial cells female-specifically express aromatase in the medaka optic tectum. *PLoS One* 8:e73663. 10.1371/journal.pone.0073663 24019933 PMC3760802

[B61] Ueda Y. Shimizu Y. Shimizu N. Ishitani T. Ohshima T. (2018). Involvement of sonic hedgehog and notch signaling in regenerative neurogenesis in adult zebrafish optic tectum after stab injury. *J. Comp. Neurol.* 526 2360–2372. 10.1002/cne.24489 30014463

[B62] Van houcke J. Mariën V. Zandecki C. Vanhunsel S. Moons L. Ayana R. (2021). Aging impairs the essential contributions of non-glial progenitors to neurorepair in the dorsal telencephalon of the Killifish *N. furzeri*. *Biorxiv* [Preprint] 10.1101/2021.02.26.433041 PMC8441397 34428340

[B63] Xiong Y. Mahmood A. Chopp M. (2013). Animal models of traumatic brain injury. *Nat. Rev. Neurosci.* 14 128–142. 10.1038/nrn3407 23329160 PMC3951995

[B64] Yang T. Dai Y. Chen G. Cui S. (2020). Dissecting the dual role of the glial scar and scar-forming astrocytes in spinal cord injury. *Front. Cell Neurosci.* 14:78. 10.3389/fncel.2020.00078 32317938 PMC7147295

[B65] Yu S. He J. (2019). Stochastic cell-cycle entry and cell-state-dependent fate outputs of injury-reactivated tectal radial glia in zebrafish. *eLife* 8:e48660. 10.7554/eLife.48660 31442201 PMC6707787

[B66] Zhao X. F. Wan J. Powell C. Ramachandran R. Myers M. G. Jr. Goldman D. (2014). Leptin and IL-6 family cytokines synergize to stimulate Muller glia reprogramming and retina regeneration. *Cell Rep.* 9 272–284. 10.1016/j.celrep.2014.08.047 25263554 PMC4194149

[B67] Zhong J. Jiang L. Cheng C. Huang Z. Zhang H. Liu H. (2016). Altered expression of long non-coding RNA and mRNA in mouse cortex after traumatic brain injury. *Brain Res.* 1646 589–600. 10.1016/j.brainres.2016.07.002 27380725

[B68] Zupanc G. K. (1999). Neurogenesis, cell death and regeneration in the adult gymnotiform brain. *J. Exp. Biol.* 202 1435–1446. 10210684 10.1242/jeb.202.10.1435

