# Supplementary material for: Differential Regenerative Capacity of the Optic Tectum of Adult Medaka and Zebrafish
Source: Front Cell Dev Biol. 2021 Jun 29;9:686755. doi: 10.3389/fcell.2021.686755 (PMC8276636; doi:10.3389/fcell.2021.686755)
Supplement: Supplementary file 2 [file Data_Sheet_1.docx]

**
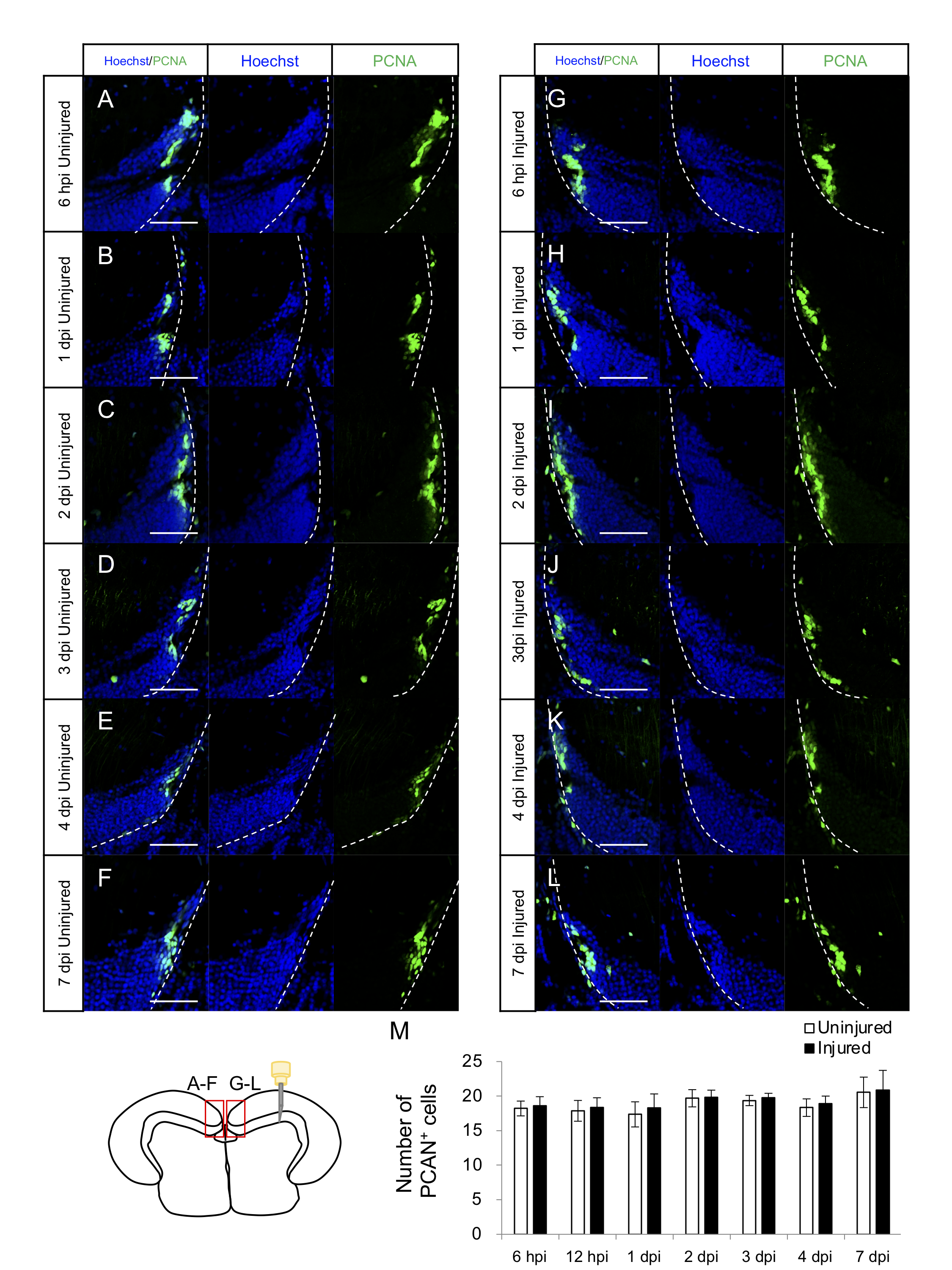
**Supplementary Figures

**Supplementary Figure 1. The proliferation of NE in the injured tectum of adult medaka.** (**A**–**F**) Representative images of proliferative NE (PCNA^+^ cells) in the uninjured hemisphere at 6 hpi, and 1, 2, 3, 4, and 7 dpi. (**G**–**L**) Representative images of proliferative NE in the injured hemisphere at 6 hpi, and 1, 2, 3, 4, and 7 dpi. Dashed lines indicate the border between optic tectum and torus longitudinalis. Scale bar: 50 µm in (**A**–**L**). Schematic drawing of stab injury in the right hemisphere of the optic tectum. (**M**) Quantification of PCNA+ cells in both uninjured and injured hemispheres at 6 hpi (*n* = 5), 12 hpi (*n* = 3), 1 dpi (*n* = 5), 2 dpi (*n* = 5), 3dpi (*n* = 4), 4 dpi (*n* = 5), 7 dpi (*n* = 4). Statistical analyses between uninjured and injured hemispheres at each time point were evaluated using paired Student’s *t*-tests.


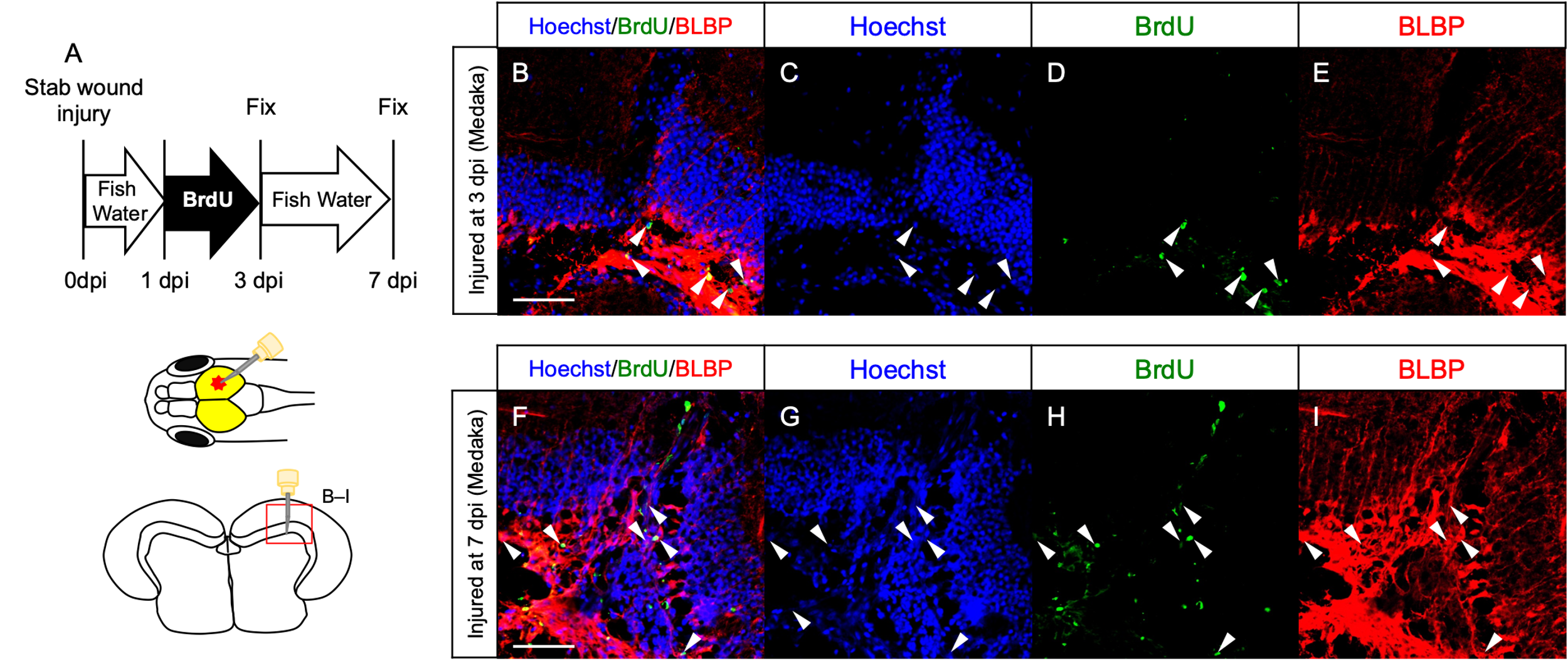


**Supplementary Figure 2. BrdU incorporation in radial glia after the tectum injury in medaka.** (**A**) Schematic drawing of BrdU treatment and stab injury in the right hemisphere of the optic tectum. (**B**–**E**) Representative images of BrdU^+^BLBP^+^ cells at 3 dpi in the medaka optic tectum. (**F**–**I**) Representative images of BrdU^+^BLBP^+^ cells at 7 dpi in the medaka optic tectum. White arrowheads indicate BrdU^+^BLBP^+^ cells in (**B**–**I**). Scale bar: 50 µm in (**B**–**I**).

**
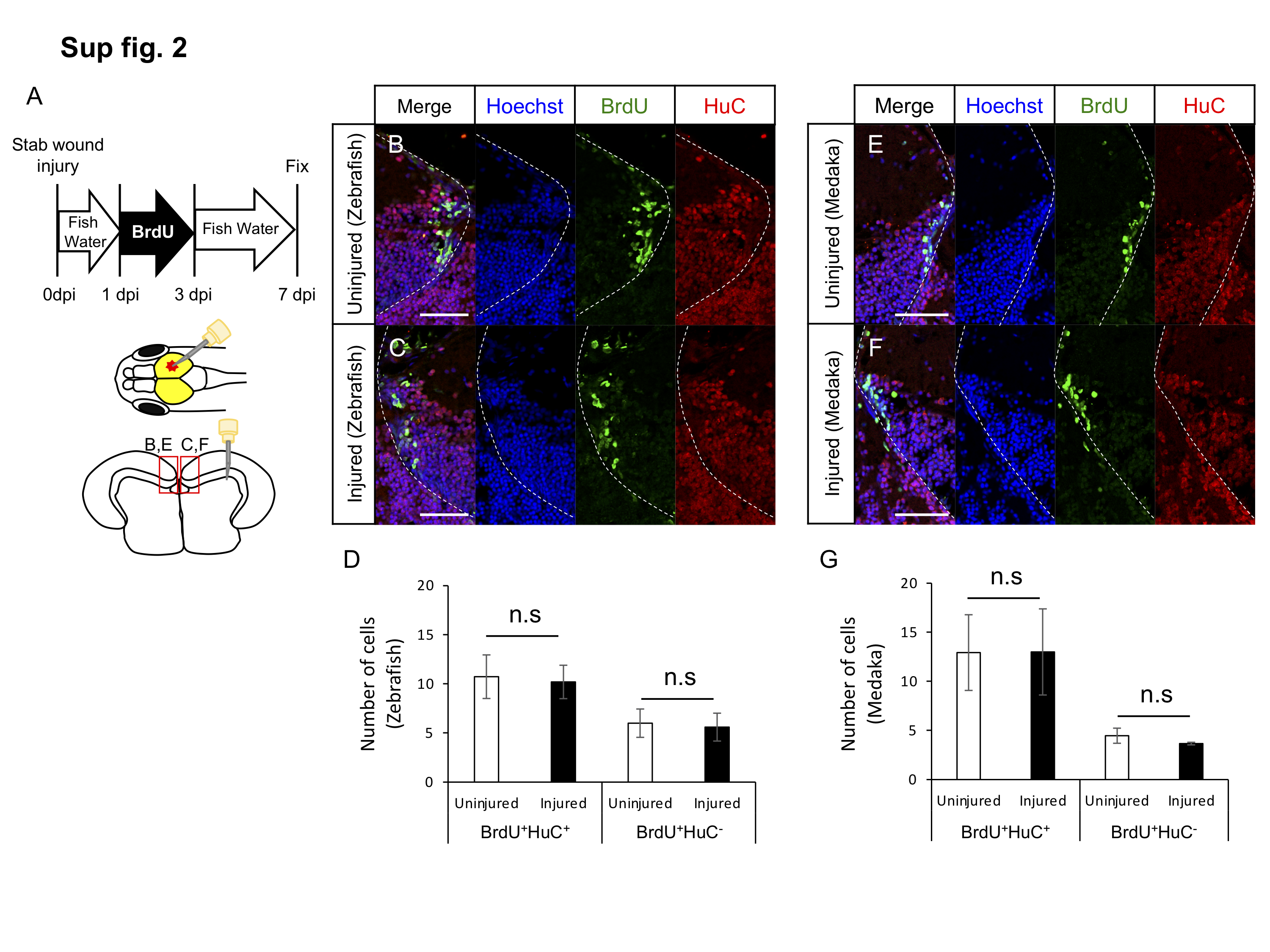
**

**Supplementary Figure 3. Generation of newborn neurons from NE after the tectum injury in medaka and zebrafish.** (**A**) Schematic drawing of BrdU treatment and stab injury in the right hemisphere of the optic tectum. (**B**–**C**) Representative images of newborn neurons (BrdU^+^HuC^+^ cells) in the uninjured (**B**) and injured (**C**) hemispheres in zebrafish. Dashed lines indicate the border between optic tectum and torus longitudinalis. Scale bar: 50 µm in (**B**–**C**). (**D**) Quantification of BrdU^+^HuC^+^ cells and BrdU^+^HuC^−^ cells in both uninjured and injured hemisphere in zebrafish (*n* = 4). Statistical analyses between uninjured and injured hemisphere were evaluated using paired Student’s t-*t*ests. (**E**–**F**) Representative images of newborn neurons (BrdU^+^HuC^+^ cells) in the uninjured (**E**) and injured (**F**) hemispheres in medaka. Dashed lines indicate the border between optic tectum and torus longitudinalis. Scale bar: 50 µm in (**E**–**F**). (**G**) Quantification of BrdU^+^HuC^+^ cells and BrdU^+^HuC^−^ cells in both uninjured and injured hemisphere in medaka (*n* = 4). Statistical analyses between uninjured and injured hemisphere were evaluated using paired Student’s *t*-tests.
